# P2Y12 inhibitor monotherapy after complex percutaneous coronary intervention: a systematic review and meta-analysis of randomized clinical trials

**DOI:** 10.1038/s41598-023-39213-3

**Published:** 2023-08-03

**Authors:** Yohei Sotomi, Yuki Matsuoka, Shungo Hikoso, Daisaku Nakatani, Katsuki Okada, Tomoharu Dohi, Hirota Kida, Bolrathanak Oeun, Akihiro Sunaga, Taiki Sato, Tetsuhisa Kitamura, Yasushi Sakata

**Affiliations:** 1 Department of Cardiovascular Medicine, Osaka University Graduate School of Medicine, 2-2 Yamadaoka, Suita, Osaka 565-0871 Japan; 2 Department of Medical Informatics, Osaka University Graduate School of Medicine, Osaka, Japan; 3 Division of Environmental Medicine and Population Sciences, Department of Social and Environmental Medicine, Graduate School of Medicine, Osaka University, Osaka, Japan

**Keywords:** Interventional cardiology, Cardiovascular diseases

## Abstract

It remains unknown whether the recent trend of short dual antiplatelet therapy (DAPT) followed by P2Y12 inhibitor monotherapy can simply be applied to patients undergoing complex percutaneous coronary intervention (PCI). We performed a systematic review and meta-analysis to evaluate P2Y12 inhibitor monotherapy vs. conventional DAPT in patients undergoing complex PCI and non-complex PCI (PROSPERO: CRD42022335723). Primary endpoint was the 1-year Net Adverse Clinical Event (NACE). Among 5,323 screened studies, six randomized trials fulfilled the eligibility criteria. A total of 10,588 complex PCI patients (5,269 vs. 5,319 patients) and 25,618 non-complex PCI patients (12,820 vs 12,798 patients) were randomly assigned to P2Y12 inhibitor monotherapy vs. conventional DAPT. In complex PCI patients, P2Y12 inhibitor monotherapy was associated with a lower risk of NACE than conventional DAPT [Odds ratio (OR) 0.76, 95% confidence interval (CI) 0.63–0.91, P = 0.003], whereas in non-complex PCI patients, P2Y12 inhibitor monotherapy was associated with a trend toward lowering the risk of NACE (OR 0.86, 95% CI 0.72–1.02, P = 0.09). This meta-analysis across randomized trials demonstrated that a strategy of short DAPT followed by P2Y12 inhibitor monotherapy reduces the risk of 1-year NACE in patients undergoing complex PCI.

## Introduction

The Academic Research Consortium (ARC) proposed the new practical definition of patients at high bleeding risk (HBR)^1^. These ARC-HBR criteria have been validated worldwide^2–6^. Although bleeding risk is now under intensive discussion in the interventional field, a thrombotic event remains an important concern for interventional cardiologists. In particular, complex PCI is considered to be an important thrombotic risk factor, and many interventional cardiologists believe that patients undergoing complex PCI should be prescribed relatively long dual antiplatelet therapy (DAPT) to prevent stent thrombosis. This was true in the era of DAPT followed by aspirin monotherapy^7^. In 2016, for example, Giustino et al. conducted a large-scale patient-level meta-analysis involving 6 randomized controlled trials^7^, and reported that compared with short-term DAPT, long-term DAPT yielded significant reductions in major adverse cardiovascular events (MACE) in the complex PCI group vs. the non-complex PCI group. However, the current mainstream of antithrombotic therapy is a short DAPT followed by P2Y12 inhibitor monotherapy. Bianco et al. performed a meta-analysis of recent trials comparing long DAPT vs. short DAPT followed by P2Y12 inhibitor monotherapy^8^. The study showed that short DAPT followed by P2Y12 inhibitor monotherapy was associated with a lower incidence of clinically relevant bleeding compared to 12-month DAPT with no significant differences in terms of cardiovascular events at 1-year follow-up. Nevertheless, it remains unknown whether this can simply be applied to patients undergoing complex PCI.

Several sub-analyses focusing on P2Y12 inhibitor monotherapy in patients with complex PCI have recently been reported^9–14^. Here, we performed a systematic review and meta-analysis to investigate the impact of short DAPT followed by P2Y12 inhibitor monotherapy on clinical outcomes in patients undergoing complex PCI.

## Methods

### Study search and eligibility criteria

We performed a systematic review and meta-analysis to evaluate P2Y12 inhibitor monotherapy vs. conventional DAPT in patients undergoing complex PCI and non-complex PCI (PROSPERO: CRD42022335723). P2Y12 inhibitor monotherapy was defined as a short DAPT (up to 3 months) followed by P2Y12 inhibitor monotherapy. Conventional DAPT was defined as a standard course of DAPT of 6–12 months followed by either aspirin or P2Y12 inhibitor monotherapy. Inclusion criteria were as follows: (1) randomized design comparing short DAPT (up to 3 months) followed by P2Y12 inhibitor monotherapy against standard DAPT, (2) use of contemporary drug-eluting stents, (3) follow-up duration ≥ 12 months, and (4) stratified analysis according to complex PCI. PubMed and Web of Science were searched from the inception of each database up to June 8th, 2022, with no restriction on language or publication status. Two investigators (YS and YM) independently assessed publications for eligibility at the title and/or abstract level, with divergences resolved by a third investigator (SH). We performed the systematic search using the following code: ((complex PCI) OR (high ischemic risk)) AND ((antiplatelet therapy) OR (monotherapy)). Because the present meta-analysis was based on data extracted from previously published research, the data and study materials are available to other researchers for purposes of reproducing the results or replicating the procedure. The analytic methods are outlined as follows.

### Data extraction and quality assessment

The following data were extracted independently by two reviewers using a standardized data abstraction form: the study year of publication, study design, inclusion and exclusion criteria, sample size, patients’ baseline characteristics, P2Y12 inhibitor used, endpoint definitions, complex PCI definitions, clinical outcomes, and follow-up duration. Quality assessment of RCTs was based on the Cochrane risk of bias tool for randomized controlled trials considering the following criteria: random sequence generation, allocation concealment, selective reporting, blinding of participants and personnel, blinding of outcome assessment, incomplete outcome data, and other sources of bias. We classified bias of the trials as low, high, or unclear.

### Study endpoints

Primary endpoint was the Net Adverse Clinical Event (NACE), which is a composite of major bleeding and major adverse cardiac and cerebrovascular events (MACCE) (Table 1) at 1-year follow-up. Secondary endpoints were bleeding endpoint (major bleeding) and all individual components of MACCE (all-cause death, myocardial infarction, stent thrombosis, and stroke) at 1-year follow-up. Since definitions of clinical endpoints were as prespecified in the individual trials, several discrepancies in definitions were present. Specifically, repeat revascularization was included in NACE in the GLOBAL LEADERS and TICO trials but not in the other trials. Stroke reported by TWILIGHT did not include hemorrhagic stroke but only ischemic stroke. Stent thrombosis was reported according to the Academic Research Consortium (ARC) definite or probable definition, except for data from GLOBAL LEADERS in which it was reported as ARC definite. Bleeding events were according to the Bleeding Academic Research Consortium (BARC) or Thrombolysis in Myocardial Infarction (TIMI) definitions. Event rates reported by TWILIGHT were at 15 months rather than 12 months. Due to limited access to the outcome data, we used these reported data as summarized in Table 1.

**Table 1:** Components of the net clinical adverse event (primary endpoint).

Study name	death	MI	Stroke	Revascularization	Stent thrombosis	Bleeding
GLOBAL LEADERS	All cause	Any	Ischemic or haemorrhagic	Any revascularization	–	BARC Type 3 or 5
MASTER DAPT	All cause	Any	Ischemic or haemorrhagic	–	–	BARC Type 3 or 5
SMART CHOICE	All cause	Any	Ischemic or haemorrhagic	–	–	BARC Type 2 or 3 or 5
STOP-DAPT2	Cardiovascular	Any	Ischemic or haemorrhagic	–	Definite	TIMI major or minor
TICO	All cause	Any	Ischemic or haemorrhagic	Target vessel revascularization	Definite or probable	TIMI major
TWILIGHT*	Cardiovascular	Any	Ischemic	–	–	BARC Type 3 or 5

*The report from the TWILIGHT did not provide the composite endpoint. We calculated the event numbers using the data of ischemic and bleeding events, but this calculation is non-hierarchical. Furthermore, event rates reported by TWILIGHT were at 15 months but not at 12 months. The numbers used in this meta-analysis is, therefore, probably overestimated to a certain degree.

### Complex PCI

The original definitions used in each trial are summarized in Table 2. Complex PCI was previously defined as including at least one of the following criteria: (1) chronic total occlusion, (2) stent length > 60 mm, (3) bifurcation with 2 stents, (4) ≥ 3 lesions treated, (5) ≥ 3 stents implanted, and (6) ≥ 3 vessels treated^7^. All trials used similar definitions, with slight differences. For the current analysis, we used the original definition in each trial. In the sub-analysis of the TICO trial only, patients were divided into high-ischemic vs. non-high-ischemic groups, in which the definition of high-ischemic included not only complex PCI but also diabetes mellitus and chronic kidney disease.

**Table 2:** Definitions of complex PCI.

Criteria	GLOBAL LEADERS	MASTER DAPT	SMART CHOICE	STOPDAPT-2	TICO	TWILIGHT
Multivessel PCI	○					
3 vessels treated		○	○	○		○
≥ 3 stents implanted	○	○	○	○	○	
≥ 3 lesion treated	○	○	○	○		○
Bifurcation with 2 stents implanted	○	○	○	○	○	○
Total stent length > 60 mm	○	○	○	○	○	○
Chronic total occlusion as the target lesion		○		○	○	○
Left main as target vessel					○	○
Graft intervention						○
Use of any atherectomy device						○

Complex PCI in each trial was defined as a procedure with at least one of the procedural criteria listed in the table.

### Statistical analysis

We performed the present systematic review and meta-analysis in accordance with the recommendations of the Cochrane Collaboration and the Preferred Reporting Items for Systematic Reviews and Meta-Analyses (PRISMA) guidelines. Analysis was performed on an intention-to-treat basis. Categorical variables are reported as percentages, and continuous variables as mean ± SD or median (interquartile range), as appropriate. A weighted average of odds ratios (OR) and 95% confidence intervals (CI) was calculated using a random-effects model, with the estimate of heterogeneity obtained using the Mantel–Haenszel method. The presence of heterogeneity among studies and subgroups was evaluated with the I^2^ statistic and the Cochran’s Q test. I^2^ values of 25%, 50%, and 75% represents mild, moderate, and severe inconsistency, respectively. A P value < 0.05 for the Cochran’s Q test was considered to indicate heterogeneity. The possibility of small study effects resulting from publication bias or other biases was examined for all endpoints by means of visual inspection of funnel plots of the ORs of individual trials against their standard errors^15,16^. We performed several sensitivity analyses to confirm the robustness of the findings. First, a sensitivity analysis was performed excluding the MASTER-DAPT trial, because the trial included patients treated with aspirin monotherapy at approximately 30%. Second, the TICO trial divided patients into high-ischemic vs non-high-ischemic risk groups, in which the high-ischemic risk included not only complex PCI but also clinical risk factors (diabetes mellitus and chronic kidney disease). In the main analysis, we used the original categorization of high-ischemic risk. Based on the published data, we could compute the event rates in patients exclusively with complex PCI only for the endpoints of NACE, MACCE, and major bleeding, and not for the other endpoints. Therefore, we performed sensitivity analyses with these computed data only for the available endpoints. Third, we conducted a sensitivity analysis without TWILIGHT trial for the NACE because NACE for complex and non-complex PCI subgroups was not reported in the trial. We calculated the event numbers as the sum of ischemic and bleeding events in a non-hierarchical manner. Lastly, since only GLOBAL LEADERS and TICO included revascularization in NACE and MACCE, we repeated the analysis without these trials. All analyses were performed using RevMan (Review Manager Version 5.3, The Cochrane Collaboration, Copenhagen, Denmark). A P value < 0.05 was considered significant. This study is registered with PROSPERO, CRD42022335723.

## Results

### Study subjects

**Figure 1:**
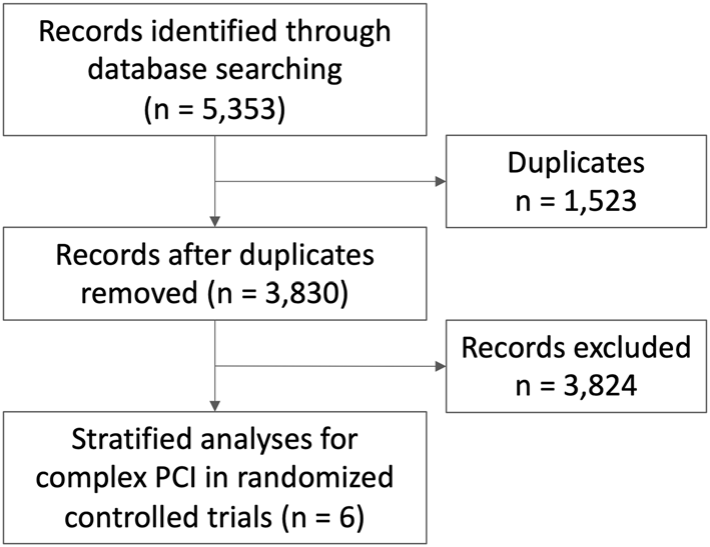
PRISMA flow diagram.

Among 5323 records screened from our search strategy, we finally included 6 studies in the meta-analysis (Fig. 1). Major characteristics of the trials are tabulated in Supplemental Table S1. Quality assessment of the trials is summarized in the Supplemental Table S2. A total of 10,588 complex PCI patients (5269 vs. 5319 patients) and 25,618 non-complex PCI patients (12,820 vs 12,798 patients) were randomly assigned to short DAPT followed by P2Y12 inhibitor monotherapy vs. conventional DAPT. Baseline characteristics of the clinical trials are summarized in Table 3. Around half of the overall population presented with acute coronary syndrome (ACS). The funnel plots are presented in Supplemental Fig. S1. Certain funnel plots exhibited asymmetrical patterns, potentially attributable to factors such as publication bias, variations in the characteristics of the included patients, and disparities in the employed P2Y12 inhibitors.

**Table 3:** Baseline characteristics.

Study name	GLOBAL leaders				SMART CHOICE				STOPDAPT-2				TWILIGHT*		TICO				MASTER DAPT			
Procedure	Complex PCI		Non-complex PCI		Complex PCI		Non-complex PCI		Complex PCI		Non-complex PCI		Complex PCI	Non-complex PCI	High-ischemic ACS		Non-high-ischemic ACS		Complex PCI		Non-complex PCI	
Treatment	P2Y12i	DAPT	P2Y12i	DAPT	P2Y12i	DAPT	P2Y12i	DAPT	P2Y12i	DAPT	P2Y12i	DAPT			P2Y12i	DAPT	P2Y12i	DAPT	P2Y12i	DAPT	P2Y12i	DAPT
Number	2283	2287	5434	5446	260	238	1235	1260	245	264	1255	1245	2342	4777	735	738	792	791	588	608	1707	1676
Age, year	65.3 (10.3)	65.2 (10.1)	64.2 (10.3)	64.3 (10.3)	64.7 (10.5)	64 (10.9)	64.6 (10.8)	64.4 (10.6)	69.2 (9.8)	69.8 (10.3)	68 (11.1)	68.9 (10.5)	66 (10.4)	64.7 (10.3)	62.9 (10.3)	63 (10.6)	58.7 (10.8)	59.4 (10.6)	76.51 (8.17)	76.78 (8.3)	75.98 (8.88)	75.66 (8.92)
BMI, kg/m2	28 (4.4)	28.1 (4.6)	28.2 (4.6)	28.2 (4.6)	24.6 (3.3)	24.8 (2.9)	24.5 (3.4)	24.7 (3.2)	24.3 (3.1)	24.6 (3.7)	24.4 (3.6)	24.2 (3.5)	28.1 (5.3)	28.8 (5.7)	24.9 (3)	25 (3.3)	24.9 (3.2)	24.8 (3.3)	27.56 (4.61)	27.58 (4.62)	27.15 (4.7)	27.39 (4.79)
Female	21.8	20.9	24.2	24.2	26.5	22.3	27.4	26.5	19.8	20.8	21.6	24.1	21.3	25.1	23.8	22.5	18.7	17.6	18.7	29.6	31.4	31.2
Diabetes mellitus	27.5	25.1	24.7	24.6	45.8	41.6	36.6	36	45.3	50	37.8	35.5	37	36.7	56.9	56.5	0	0	34.4	33.4	32.3	34.7
Current smoking	26.9	26.5	25.4	26.4	25.8	25.2	29	24.4	22.9	20.8	27.3	20.6	20.6	22.3	N.A	N.A	N.A	N.A	8	6.4	10.8	8.7
Hypertension	74.5	73	73.5	73.4	68.1	68.5	60.3	60	75.9	76.5	73.2	73.4	71.2	73	60	60.8	40.3	42	80.4	77	75.7	78.7
Dyslipidemia	69.8	71.2	69.2	69.6	44.2	45	45.3	45.7	78	80.3	73.7	73.6	58.2	61.6	63.7	62.1	57.6	58.7	71.4	66.3	65.7	68.7
CKD (eGFR < 60)	14.1	14	13.8	13.3	6.2	5	2.3	3.3	45.3	42	39.2	40.1	18.1	16.1	39.7	44.4	0	0	22.3	20.1	16.8	20
Previous MI	20.9	21.8	23.5	24.1	3.5	3.8	4.3	4.4	18.4	17.1	12.9	12.4	28.7	28.6	4.5	4.3	3.9	2.1	21.1	23.8	18.2	17
ACS	48.6	48.6	46.4	46.2	54.6	61.3	58.9	57.6	34.3	30.3	38.3	40.4	63.6	65.4	100	100	100	100	48.1	47.3	49.5	47.4

Data are expressed as mean (standard deviation) or percentage (%).

*ACS* acute coronary syndrome, *BMI* body mass index, *CKD* chronic kidney disease, *DAPT* dual antiplatelet therapy, *MI* myocardial infarction, *P2Y12i* P2Y12 inhibitor, *N.A.* not available.

*The report from the TWILIGHT did not provide the data stratified by treatment arm.

^†^Only in the TICO trial, patients were divided into high-ischemic ACS vs. non-high-ischemic ACS. The definition of high-ischemic ACS included not only complex PCI but also diabetes mellitus and chronic kidney disease.

### Impact of P2Y12 inhibitor monotherapy in complex PCI and non-complex PCI

#### Primary endpoint

**Figure 2:**
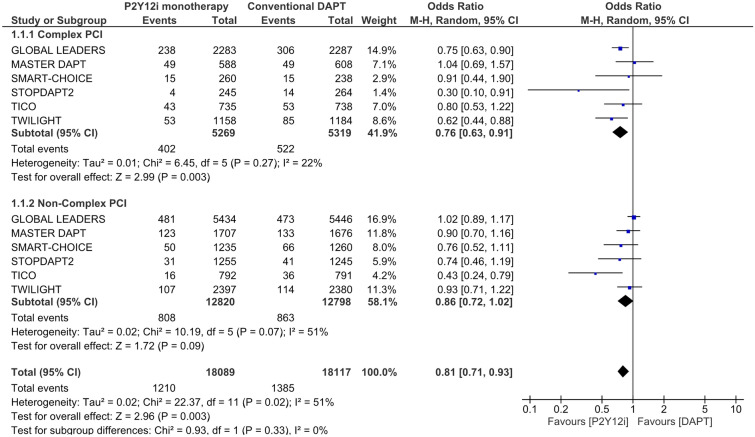
Risk estimation for the primary endpoint: net adverse clinical events.

Results of the primary endpoint are summarized in Fig. 2. In complex PCI patients, P2Y12 inhibitor monotherapy was associated with a lower risk of NACE than conventional DAPT [7.6% vs. 9.8%, Odds ratio (OR) 0.76, 95% confidence interval (CI) 0.63–0.91, P = 0.003], whereas in non-complex PCI patients, P2Y12 inhibitor monotherapy was associated with a trend toward lowering the risk of NACE (6.3% vs. 6.7%, OR 0.86, 95% CI 0.72–1.02, P = 0.09). No heterogeneity was found between complex and non-complex PCI patients (I^2^ = 0%, P = 0.33).

#### Bleeding endpoint

**Figure 3:**
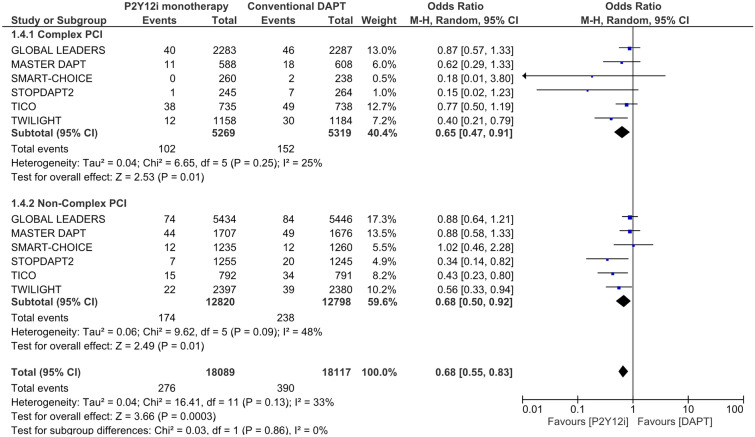
Risk estimation for the bleeding endpoint: major bleeding.

Results of bleeding event are summarized in Fig. 3. Both in complex and non-complex PCI patients, P2Y12 inhibitor monotherapy was associated with a lower risk of major bleeding than conventional DAPT (complex PCI, OR 0.65, 95% CI 0.47–0.91, P = 0.01; non-complex PCI, OR 0.68, 95% CI 0.50–0.92, P = 0.01). The results were consistent across complex and non-complex PCI patients (I^2^ = 0%, P = 0.86).

### MACCE and its individual components

**Figure 4:**
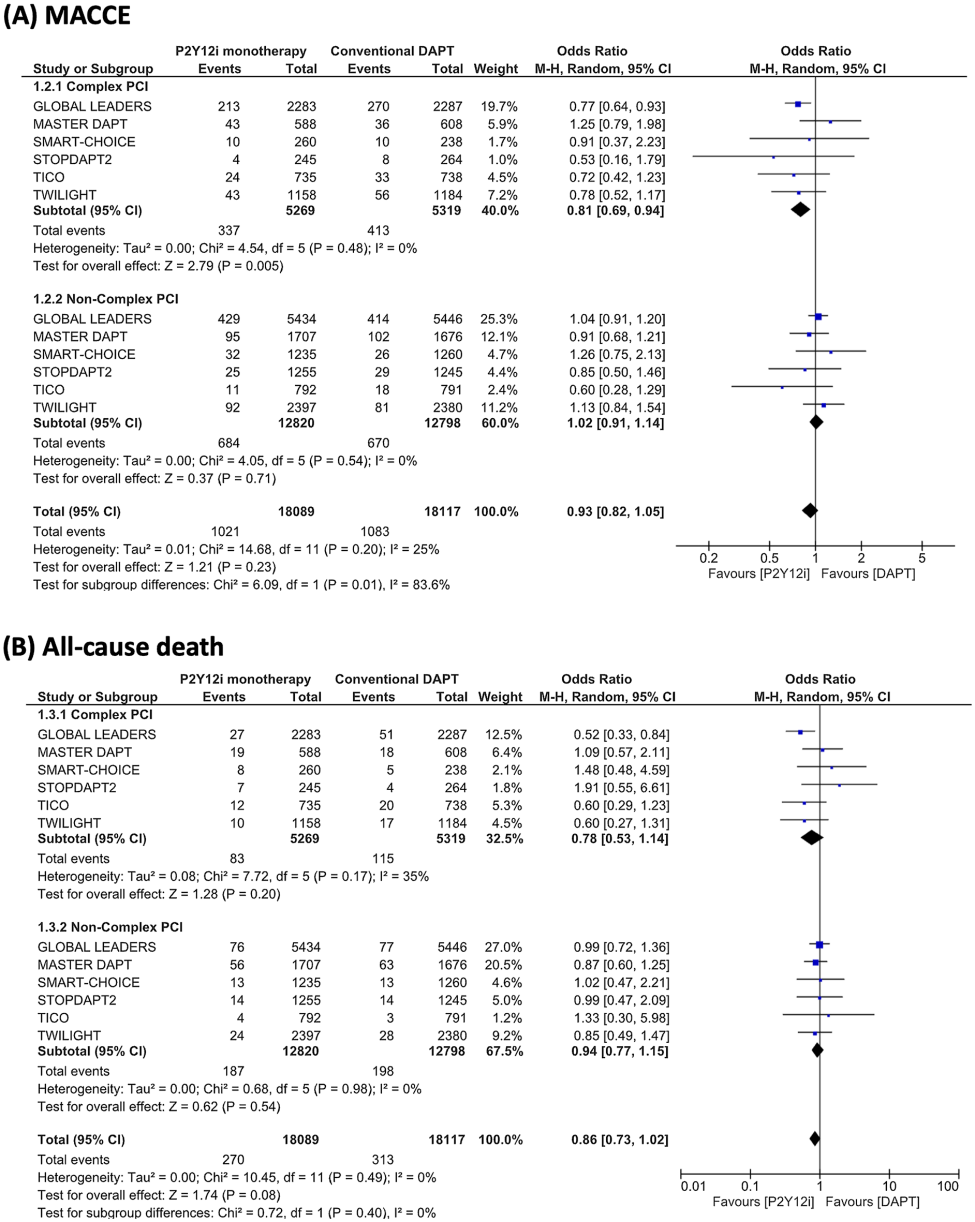

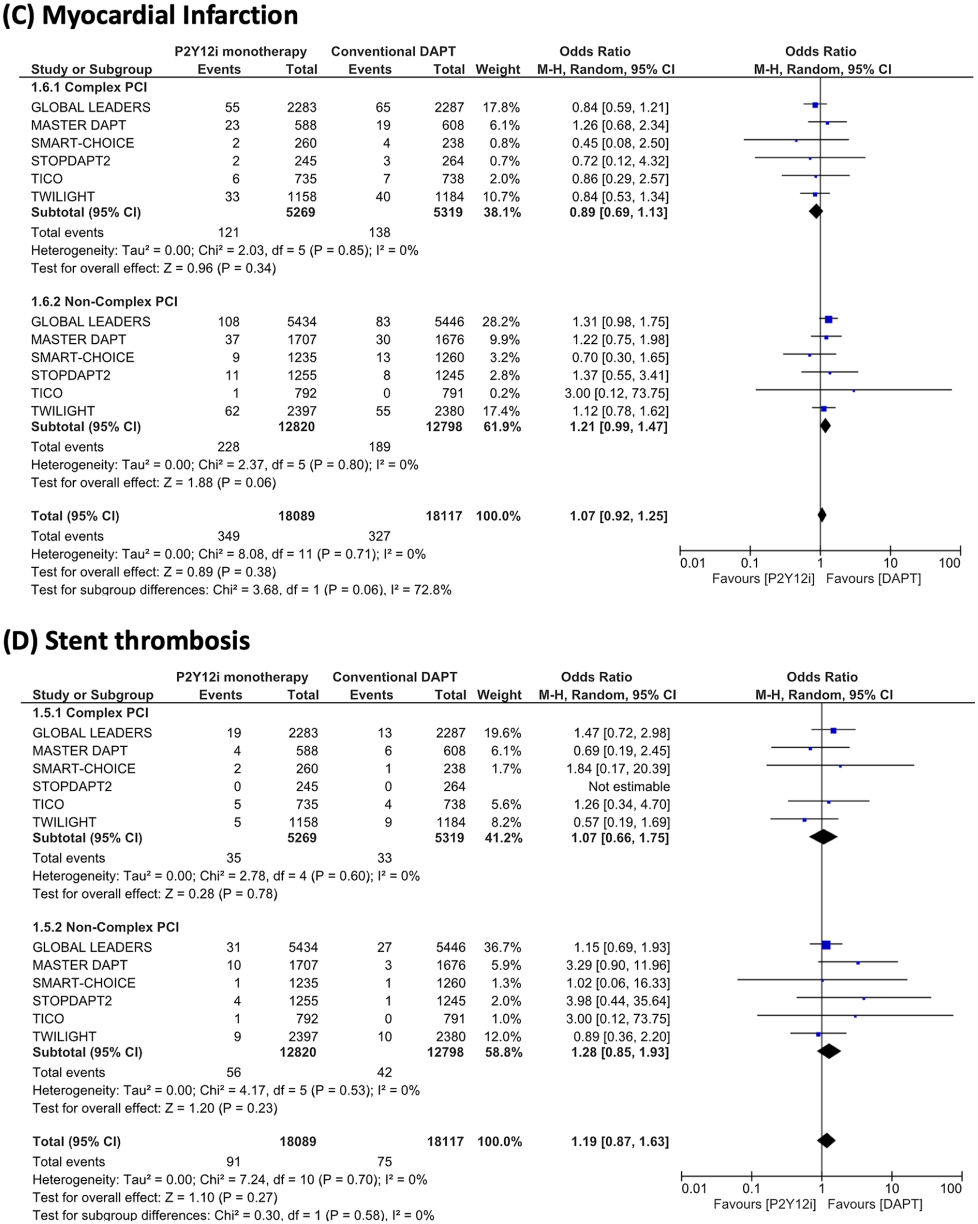

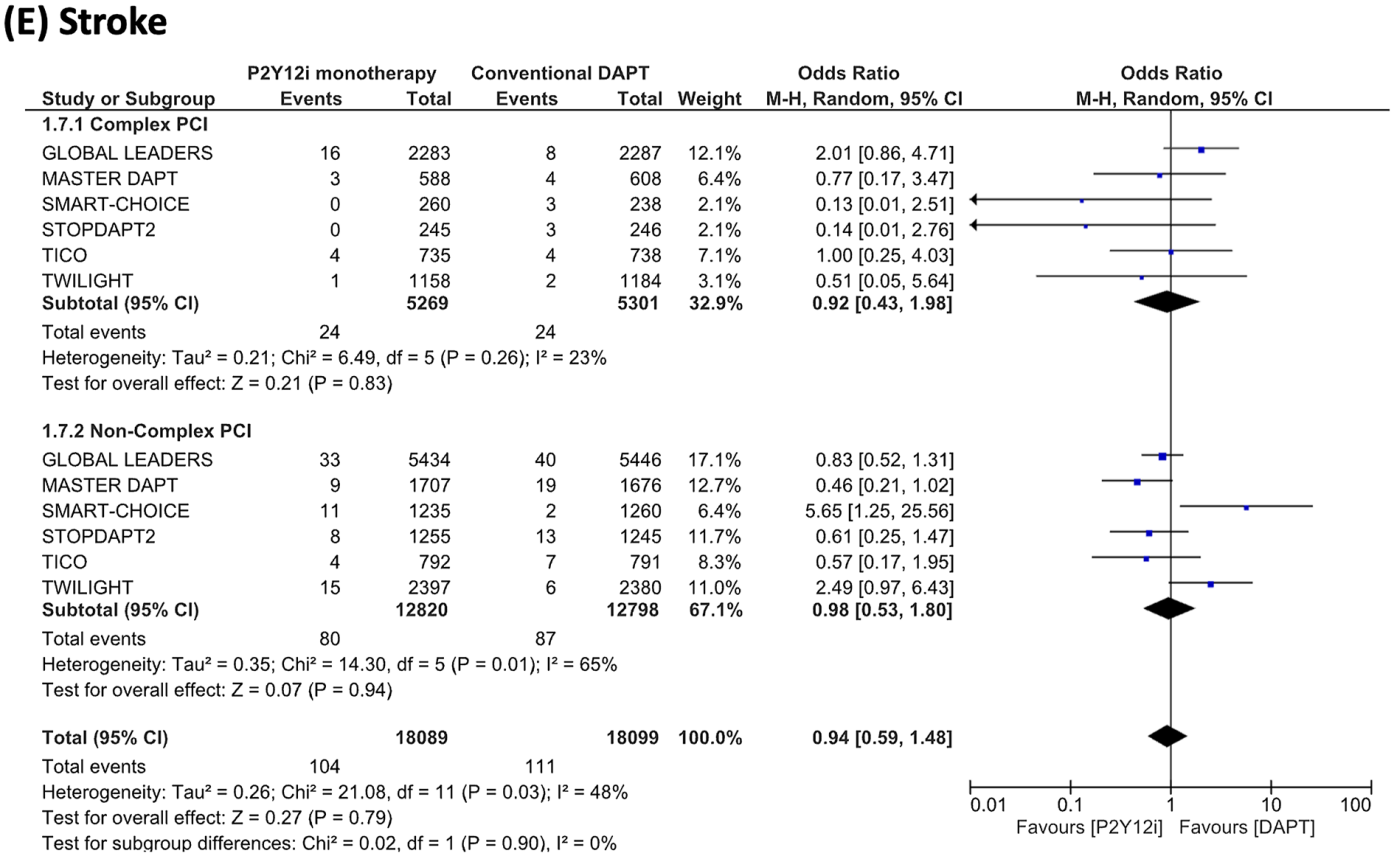
Risk estimations for MACCE and its individual components. Risk estimations for MACCE and its individual components are shown for (**A**) major adverse cardiac and cerebrovascular events (MACCE), (**B**) all-cause death, (**C**) myocardial infarction (**D**) stent thrombosis, and (**E**) stroke.

Results of the ischemic endpoint are summarized in Fig. 4. P2Y12 inhibitor monotherapy was associated with a lower risk of MACCE in complex PCI patients (OR 0.81, 95% CI 0.69–0.94, P = 0.005), but was not associated with a lower risk in non-complex PCI patients (OR 1.02, 95% CI 0.91–1.14, P = 0.71) (I^2^ = 83.6%, P = 0.01). The endpoints of all-cause death, myocardial infarction, stent thrombosis, and stroke did not differ between P2Y12 inhibitor monotherapy and conventional DAPT in both complex PCI and non-complex PCI patient groups.

### Sensitivity analyses

All these analyses were repeated following exclusion of the MASTER-DAPT trial as a sensitivity analysis. Overall, results were found to be consistent (Supplemental Figs. S2, S3, S4). Results of another sensitivity analysis with computed results of pure population with complex PCI in the TICO trial were also totally consistent with the main analysis (NACE, Supplemental Fig. S5; MACCE, Supplemental Fig. S6; Major bleeding, Supplemental Fig. S7). Another sensitivity analysis for NACE without TWILIGHT trial is illustrated in Supplemental Fig. S8. The result was consistent with the main analysis. The final sensitivity analyses for NACE and MACCE, excluding GLOBAL LEADERS and TICO, are presented in Supplemental Figs. S9 and S10, respectively. While the point estimates for NACE suggested a beneficial impact of P2Y12 inhibitor monotherapy in individuals undergoing complex PCI and non-complex PCI, these findings did not attain statistical significance. Moreover, there were no notable disparities in MACCE rates between the P2Y12 inhibitor monotherapy and conventional DAPT groups within both the complex PCI and non-complex PCI patient cohorts.

## Discussion

We conducted this meta-analysis to investigate whether a short DAPT followed by P2Y12 inhibitor monotherapy influences clinical outcome in patients undergoing complex or non-complex PCI. Compared with conventional DAPT, short DAPT followed by P2Y12 inhibitor monotherapy was (1) associated with a lower risk of 1-year NACE in complex PCI patients; (2) associated with a trend toward lowering the risk of NACE in non-complex PCI patients; (3) associated with a lower risk of 1-year major bleeding both in complex and non-complex PCI patients; and (4) associated with a lower risk of 1-year MACCE in complex PCI patients only, and not in non-complex PCI patients.

The most recent large-scale analysis on this topic was reported by Giustino et al.^7^, who found that that prolonged (12–24 months) DAPT reduced major adverse cardiac events and coronary thrombotic events compared with short (3–6 months) DAPT after complex PCI in the patient-level pooled analysis of 6 RCTs^7^. Accordingly, European Society of Cardiology and Japanese Circulation Society guidelines have suggested that complex PCI is a risk factor of stent-driven recurrent ischemic events^17,18^. Given this background, many interventional cardiologists seem reluctant to choose a short DAPT strategy in patients undergoing complex PCI despite the recent favorable data of P2Y12 inhibitor monotherapy. However, recent sub-analyses from the GLOBAL LEADERS, STOPDAPT2, TICO, TWILIGHT, SMART-CHOICE, and MASTER-DAPT trials showed consistent and somewhat unexpected results for interventional cardiologists^9–14^, namely that the short DAPT strategy works even better in complex PCI patients than in non-complex PCI patients. To confirm the robustness of this finding, we conducted the current meta-analysis. Along the same line with the recent meta-analyses without MASTER-DAPT trial^19,20^, we found that short DAPT followed by P2Y12 inhibitor monotherapy was associated with a lower risk of NACE, MACCE, and major bleeding than conventional DAPT in complex PCI patients. In non-complex PCI patients, P2Y12 inhibitor monotherapy was associated with a trend toward lowering the risk of NACE and a lower risk of major bleeding, but not with MACCE. The discrepancy between the previous and this meta-analysis might be explained by the use of P2Y12 inhibitor monotherapy after DAPT in the recent trials over aspirin used in the previous trials^7^. The recent antiplatelet regimen with an initial short-term duration of DAPT to prevent stent-related thrombotic events followed by a long-term course of a potent P2Y12 inhibitor alone has been expected to reduce the excess of aspirin-related bleeding without reducing anti-ischemic efficacy. The antiplatelet effect of P2Y12 inhibitor monotherapy might be strong enough to afford protection against ischemic events regardless of PCI complexity.

It has been reported that patients with complex PCI were at higher bleeding risk than those without because of overlapping risk factors^9,17,18^. Indeed, in this meta-analysis, patients undergoing complex PCI more frequently experienced major bleeding events than those undergoing non-complex PCI [2.40% (254/10,588) vs 1.61% (412/25,618), Fig. 3]. This may be because the more complex CAD a patient has, the more comorbidity burdens the patient is likely to have. Complex CAD is attributed to such comorbidities, but these often exist as bleeding risks at the same time. Patients with multiple HBR criteria have a higher bleeding risk than those with a single HBR criterion^21–23^. Therefore, if we consider the balance between bleeding and thrombotic events, bleeding risk may be particularly weighted in complex PCI patients.

It is worth mentioning that the utilization of P2Y12 inhibitor monotherapy was associated not only with a decreased risk of bleeding but also with a reduction in MACCE occurrence in patients undergoing complex PCI. However, it is important to note that this finding, which exhibited consistency across the trials included in this meta-analysis (I^2^ = 0%), was primarily driven by the outcomes of the GLOBAL LEADERS and TICO trials, wherein repeat revascularization was considered within the composite endpoint. Our sensitivity analysis, excluding these trials, revealed an insignificant impact of P2Y12 inhibitor monotherapy, in contrast to the main findings. Similarly, a meta-analysis conducted by Gragnano et al., employing pooled patient-level data, also failed to demonstrate a significant effect of short-DAPT followed by P2Y12 inhibitor monotherapy in relation to ischemic composite endpoints (comprising all-cause death, myocardial infarction, and stroke) when compared to the conventional DAPT strategy in both complex and non-complex PCI patients (complex PCI, hazard ratio [HR] 0.87, 95% confidence interval [CI] 0.64–1.19; non-complex PCI, HR 0.91, 95% CI 0.76–1.09)^24^. Therefore, while this antithrombotic regimen may provide protection against bleeding complications, its impact on severe ischemic events, as suggested by the individual trial results, may not be statistically significant.

### Clinical implications

The most important message of this meta-analysis is that PCI complexity does not justify a more prolonged course of DAPT, or rather that it warrants short DAPT followed by P2Y12 inhibitor monotherapy. This is supported by the report from Urban et al. showing that a complex PCI procedure was significantly associated with increased risk of both thrombotic and bleeding events^25^. However, the generalizability of the current findings should be carefully considered. First, the applicability of our results to ACS patients should be carefully considered. Although a recent meta-analysis of 9 RCTs consisting of 25,907 ACS patients suggested that 1–3 months of DAPT has similar efficacy in preventing ischemic events with reduced bleeding risk compared with 6 to 12 months of DAPT^26^, ACS with complex PCI is likely to be a strong ischemic risk factor. Second, ticagrelor or clopidogrel were mainly evaluated in the included trials. However, this meta-analysis did not address any preferred P2Y12 inhibitors. Further research should investigate which P2Y12 inhibitor is the drug of choice after discontinuing DAPT in complex PCI patients. Third, although the extent and complexity of complex PCI was not found to be associated with MACCE (all-cause death, MI, or stroke) in the TWILIGHT trial^10^, the relationship between PCI complexity and clinical events could not be precisely assessed in this study-level meta-analysis. Patient-level meta-analysis will provide important insights into this point.

### Study limitations

Several limitations of this meta-analysis should be acknowledged. First, the study designs of the 6 included trials differed, including in their use of placebo, choice of P2Y12 inhibitor, use of oral anticoagulation, proportion of acute coronary syndrome, and timing of randomization. Second, the study was conducted as a study-level meta-analysis, and not as a patient-level meta-analysis. Third, the definition of primary endpoint differed slightly among the included trials (Table 1); in particular, GLOBAL-LEADERS and TICO included revascularization in the endpoint, resulting in relatively larger event numbers than the other trials. Stroke reported from TWILIGHT included only ischemic stroke, and not hemorrhagic stroke. The MASTER DAPT trial included approximately 30% patients taking aspirin monotherapy. This small proportion of aspirin monotherapy may have resulted in noise in the analysis, albeit that the population with aspirin monotherapy is markedly limited compared to the overall population. To confirm the robustness of the findings, we performed several sensitivity analyses and found that the overall results were consistent with the main results. Fourth, because the report from the TWILIGHT did not provide NACE, we computed the event numbers using the data for ischemic and bleeding events. However, this calculation was non-hierarchical. The numbers used in this meta-analysis are therefore likely overestimated to a certain degree. Fifth, the TICO trial divided patients into high-ischemic vs non-high-ischemic risk groups, wherein the high-ischemic risk included not only complex PCI but also clinical risk factors (diabetes mellitus and chronic kidney disease). This may have somewhat influenced the results, although our sensitivity analysis demonstrated consistent findings. Lastly, around 10% of patients in the standard DAPT arm in MASTER-DAPT trial received short DAPT and subsequent P2Y12 inhibitor monotherapy (with complex PCI, 11.8%; with non-complex PCI, 9.1% at 3 months visit)^14^. Although there were instances of crossovers across all the studies, the specific details regarding these crossovers and their distribution within the subgroups of complex PCI and non-complex PCI were not available.

## Conclusions

This meta-analysis across randomized trials demonstrated that a strategy of short DAPT followed by P2Y12 inhibitor monotherapy reduces the risk of NACE in patients undergoing complex PCI. PCI complexity does not justify a more prolonged course of DAPT, or rather warrants short DAPT followed by P2Y12 inhibitor monotherapy.

### Supplementary Information

Supplementary Information.

## Data Availability

The authors confirm that the data supporting the findings of this study are available within the referenced articles and their supplementary materials.
