# Supplementary material for: P2Y12 inhibitor monotherapy after complex percutaneous coronary intervention: a systematic review and meta-analysis of randomized clinical trials
Source: Sci Rep. 2023 Aug 3;13:12608. doi: 10.1038/s41598-023-39213-3 (PMC10400615; doi:10.1038/s41598-023-39213-3)
Supplement: Supplementary file 1 — Supplementary Information. [file 41598_2023_39213_MOESM1_ESM.docx]

**Supplemental Materials**

**Supplemental Figures**

- Supplemental Figure 1. Funnel plots
- Supplemental Figure 2. Risk estimation for the primary endpoint: net adverse clinical endpoints (analysis without MASTER-DAPT trial)
- Supplemental Figure 3. Risk estimation for the bleeding endpoint: major bleeding (analysis without MASTER-DAPT trial)
- Supplemental Figure 4. Risk estimations for MACCE and its individual components (analysis without MASTER-DAPT trial)
- Supplemental Figure 5. Risk estimation for the primary endpoint: net adverse clinical endpoints (pure population with complex PCI in TICO)
- Supplemental Figure 6. Risk estimation for the bleeding endpoint: major bleeding (pure population with complex PCI in TICO)
- Supplemental Figure 7. Risk estimations for MACCE and its individual components (pure population with complex PCI in TICO)
- Supplemental Figure 8. Risk estimations for NACE without TWILIGHT trial
- Supplemental Figure 9. Risk estimations for NACE without GLOBAL LEADERS and TICO
- Supplemental Figure 10. Risk estimations for MACCE without GLOBAL LEADERS and TICO

**Supplemental Tables**

- Supplemental Table 1. Risk of bias assessment
- Supplemental Table 2. Study characteristics

**Supplemental Figure 1. Funnel plots**

Funnel plots are shown for (**A**) Net Adverse Clinical Event (NACE), (**B**) major adverse cardiac and cerebrovascular events (MACCE), (**C**) major bleeding (**D**) all-cause death, (**E**) myocardial infarction (**F**) stent thrombosis, and (**G**) stroke. Certain funnel plots exhibited asymmetrical patterns, potentially attributable to factors such as publication bias, variations in the characteristics of the included patients, and disparities in the employed P2Y12 inhibitors.

**
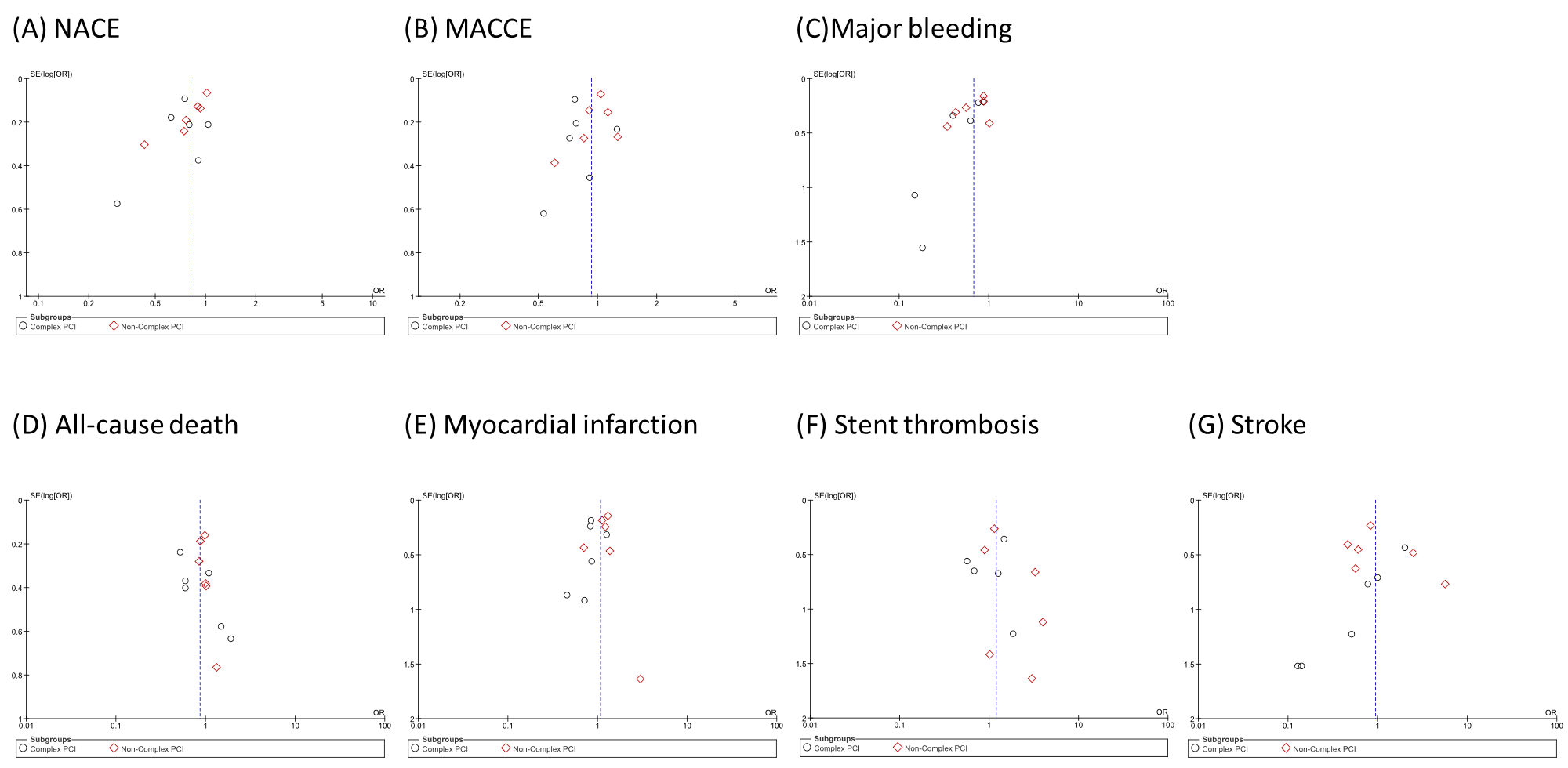
**

**Supplemental Figure 2. Risk estimation for the primary endpoint: net adverse clinical endpoints (analysis without MASTER-DAPT trial)**

**
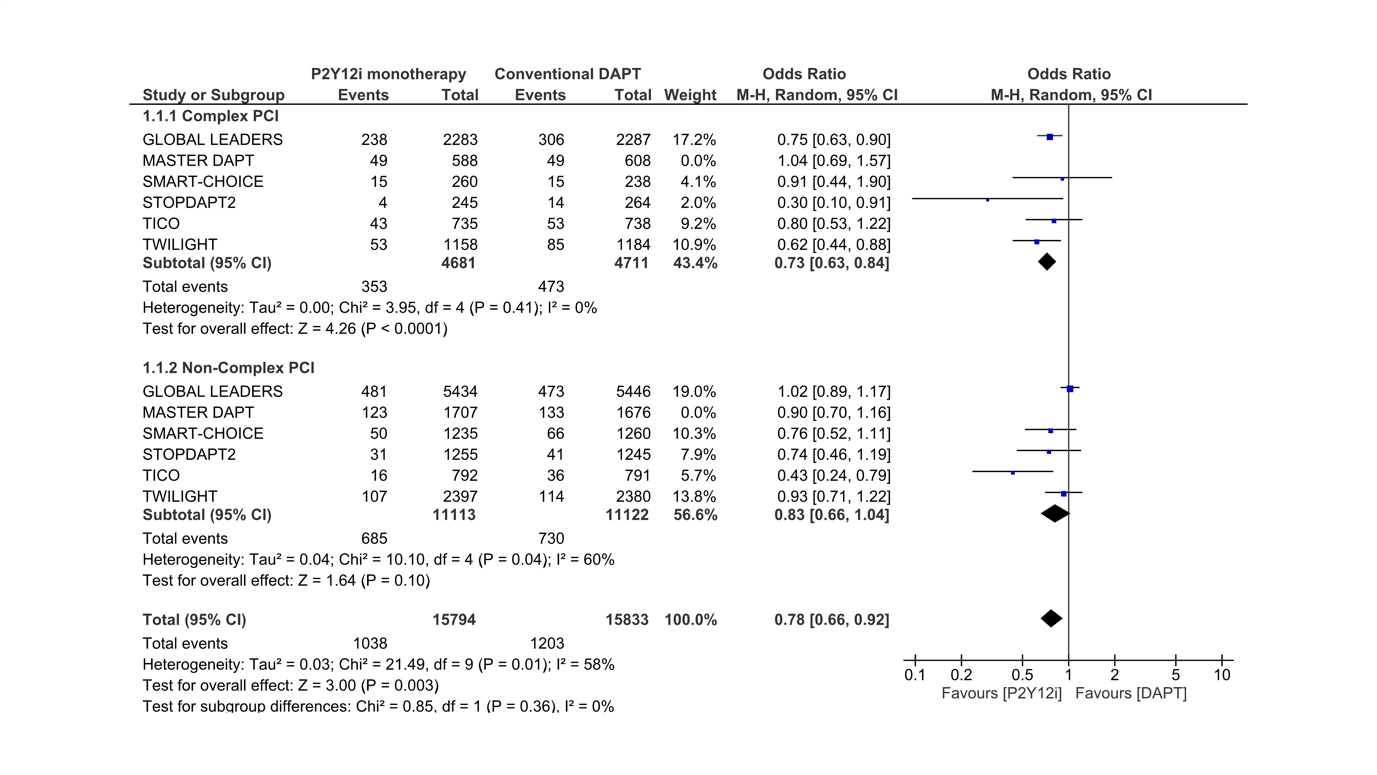
**

**Supplemental Figure 3. Risk estimation for the bleeding endpoint: major bleeding (analysis without MASTER-DAPT trial)**

**
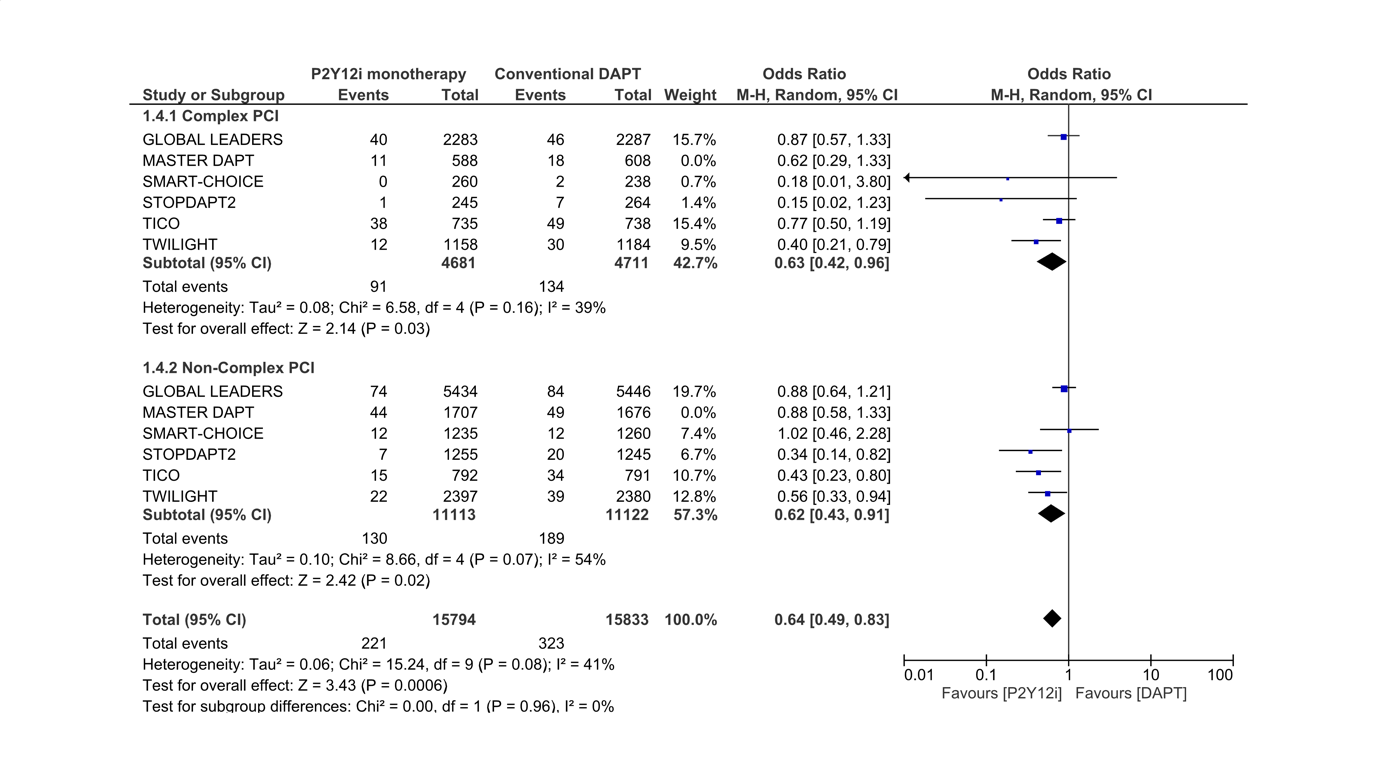
**

**Supplemental Figure 4. Risk estimations for MACCE and its individual components (analysis without MASTER-DAPT trial)**

**
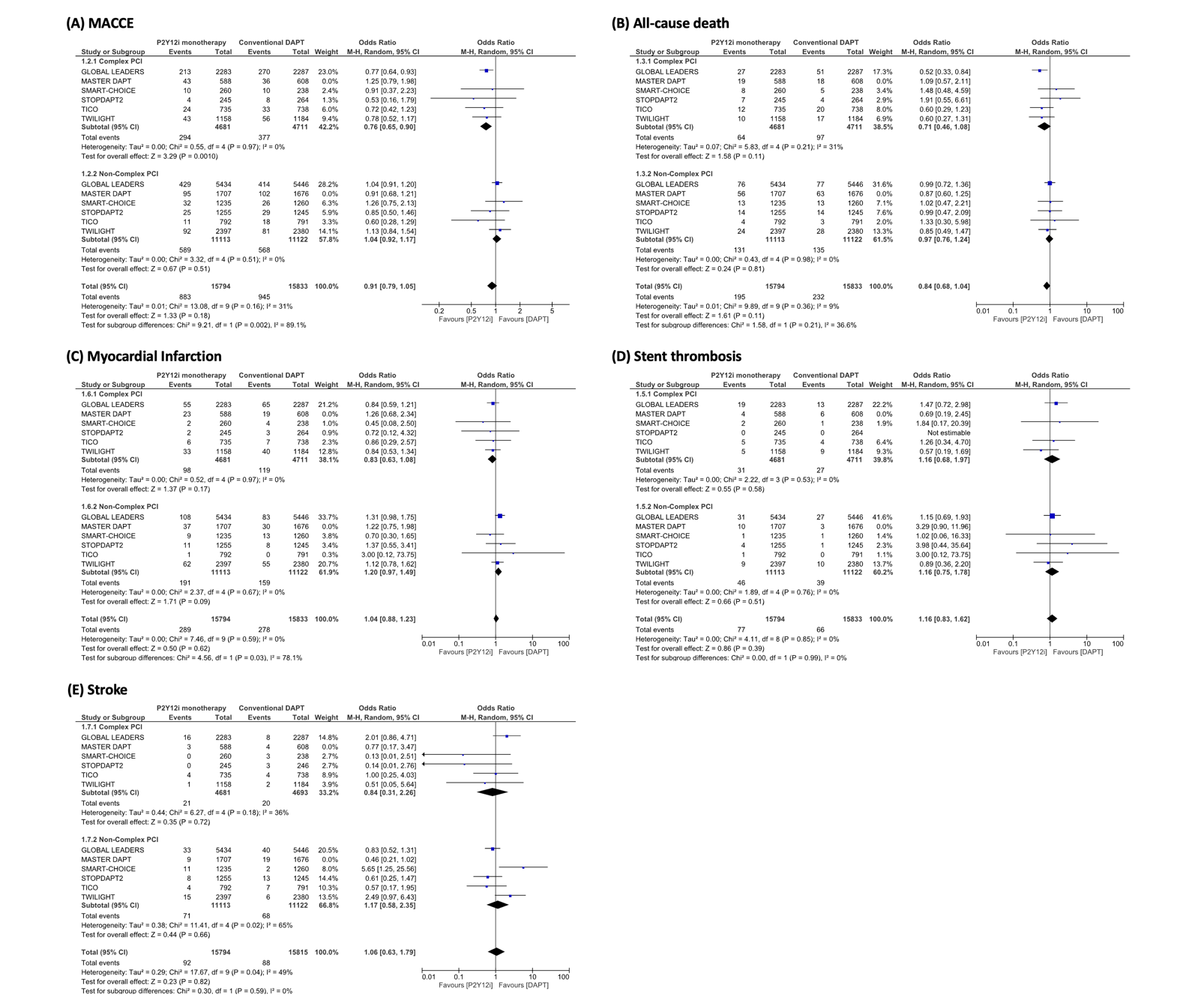
**

Risk estimations for MACCE and its individual components are shown for (A) major adverse cardiac and cerebrovascular events (MACCE), (B) all-cause death, (C) myocardial infarction (D) stent thrombosis, and (E) stroke.

**Supplemental Figure 5. Risk estimation for the primary endpoint: net adverse clinical endpoints (pure population with complex PCI in TICO)**


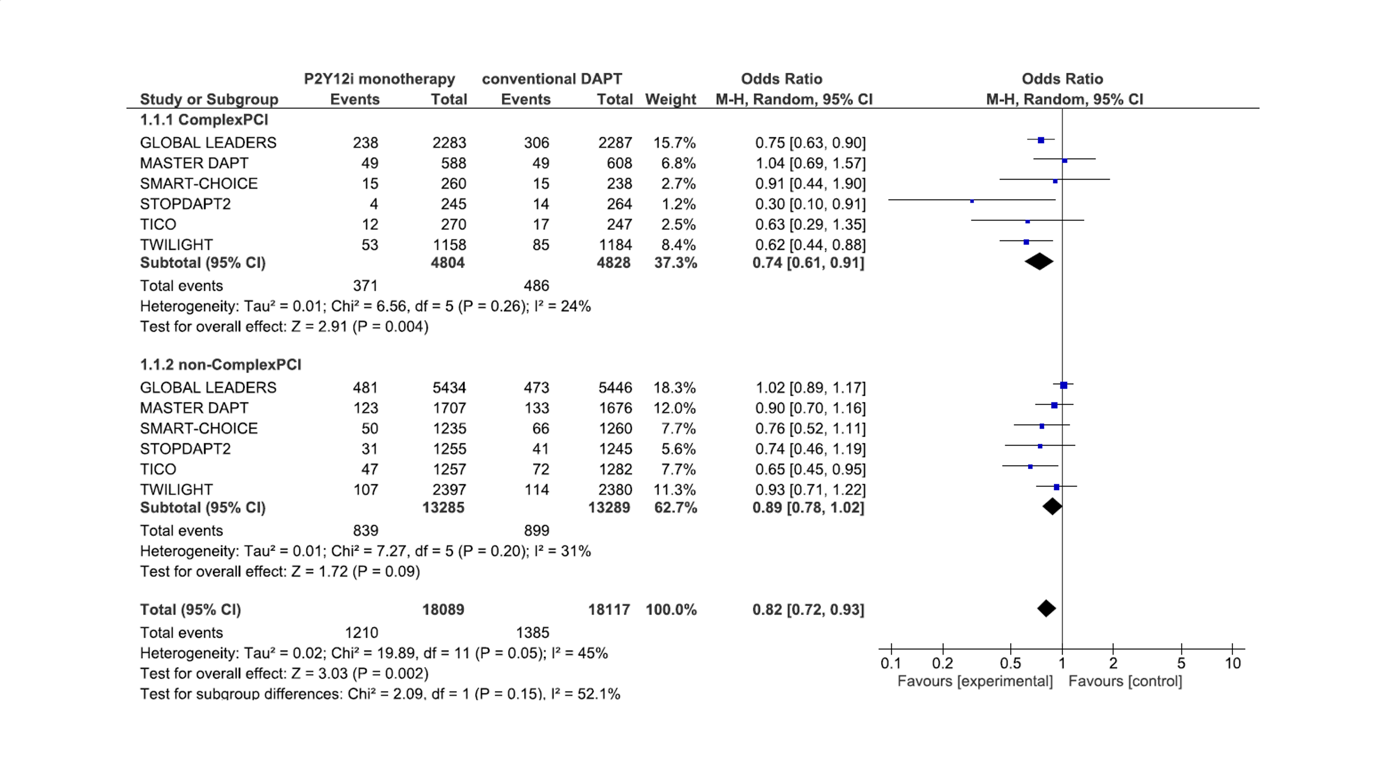


**Supplemental Figure 6. Risk estimation for the bleeding endpoint: major bleeding (pure population with complex PCI in TICO)**


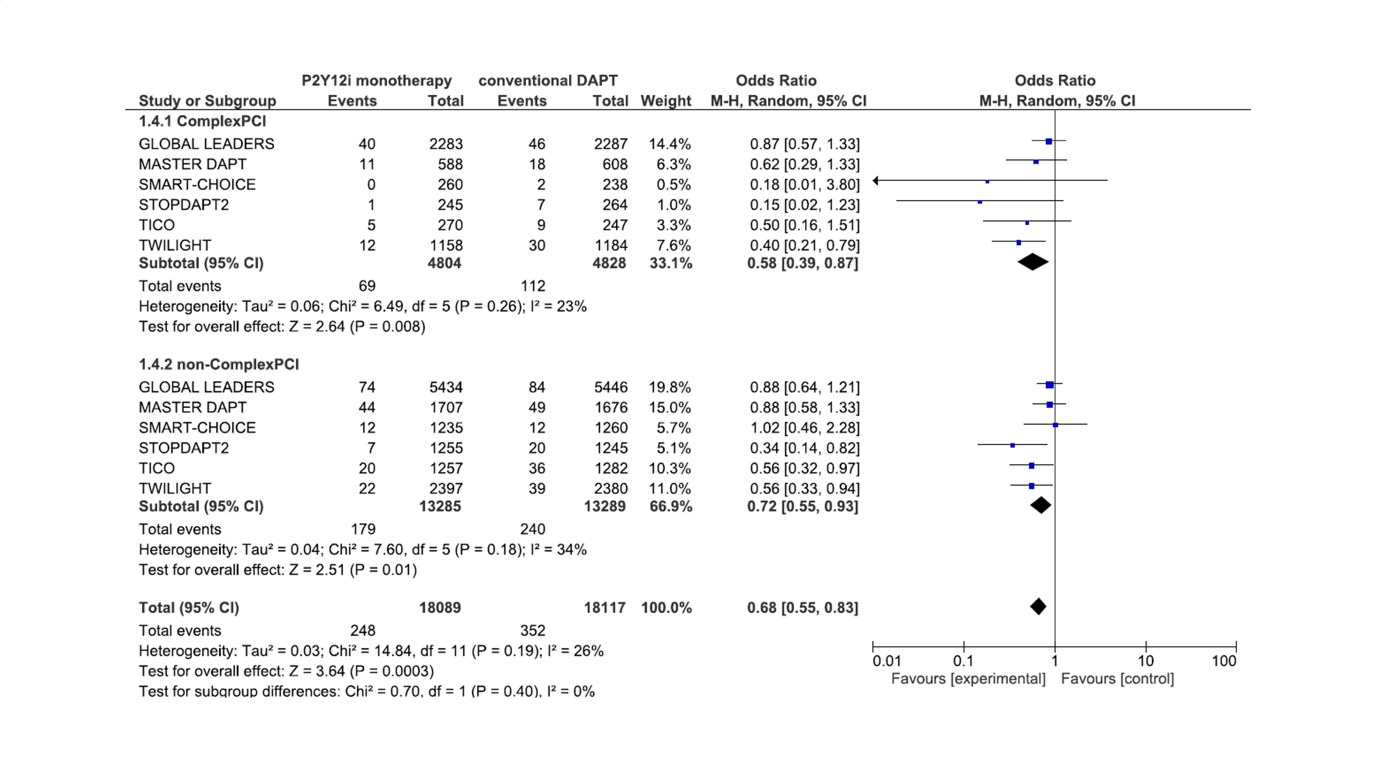


**Supplemental Figure 7. Risk estimations for MACCE (pure population with complex PCI in TICO)**


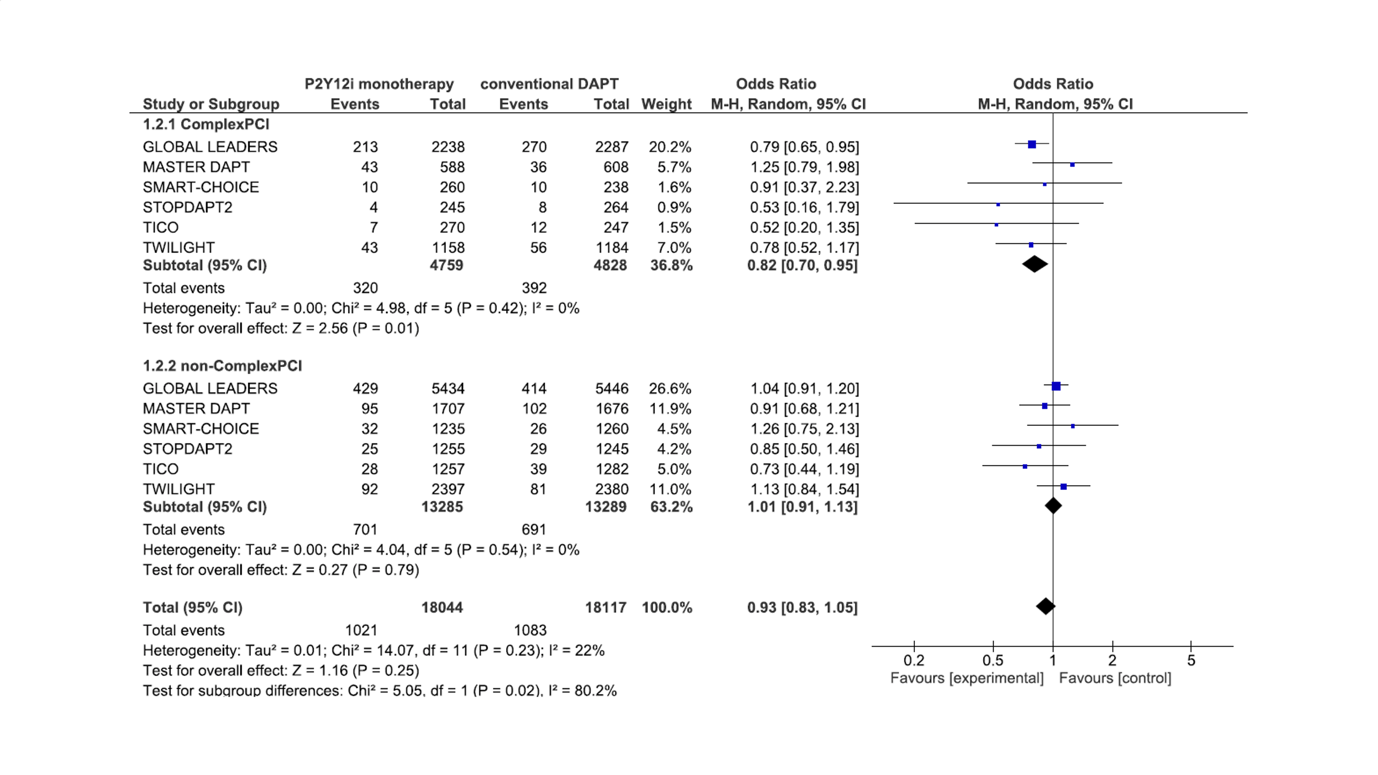


**Supplemental Figure 8. Risk estimations for NACE without TWILIGHT trial**


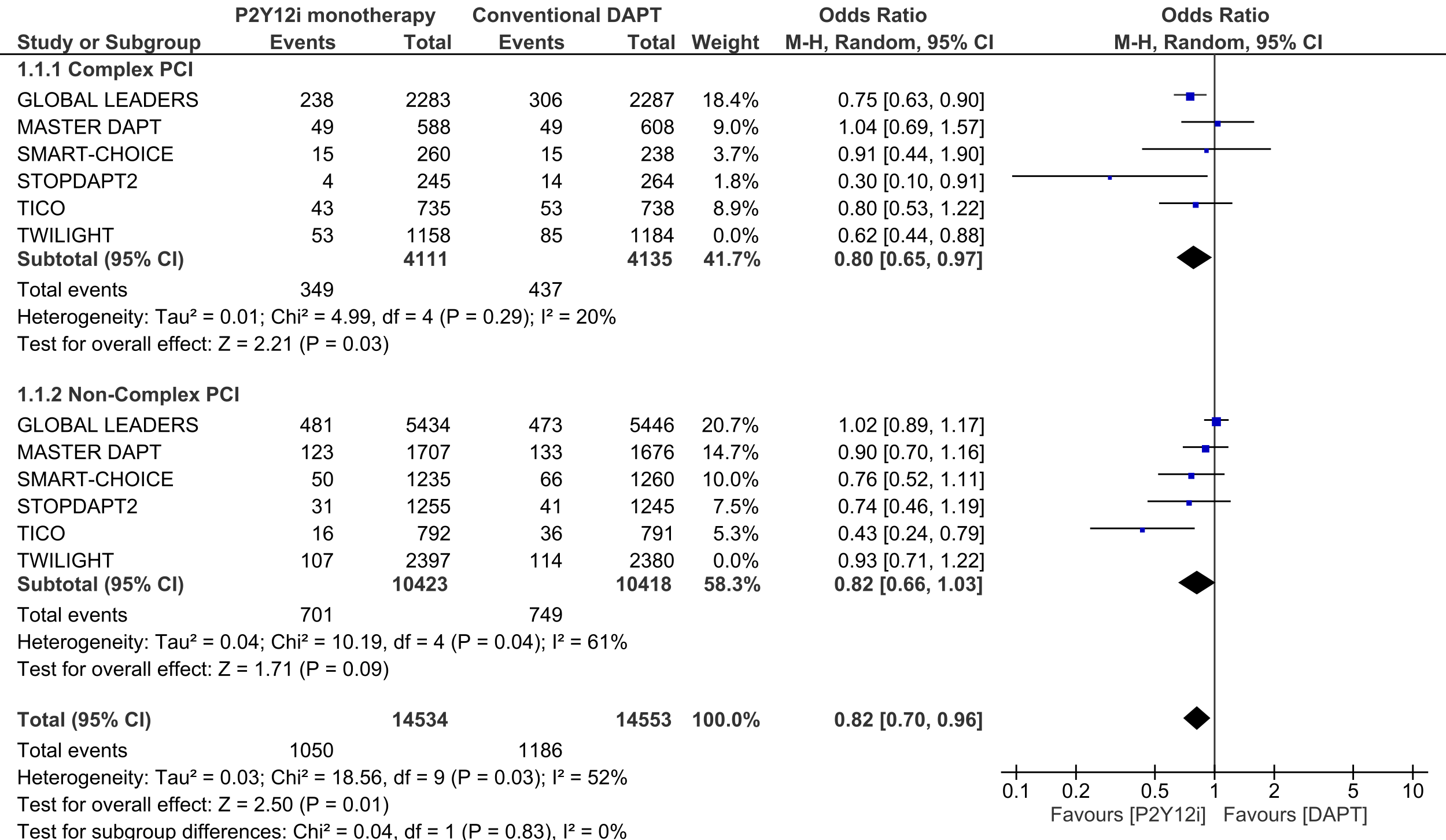


**Supplemental Figure 9. Risk estimations for NACE without GLOBAL LEADERS and TICO**

**
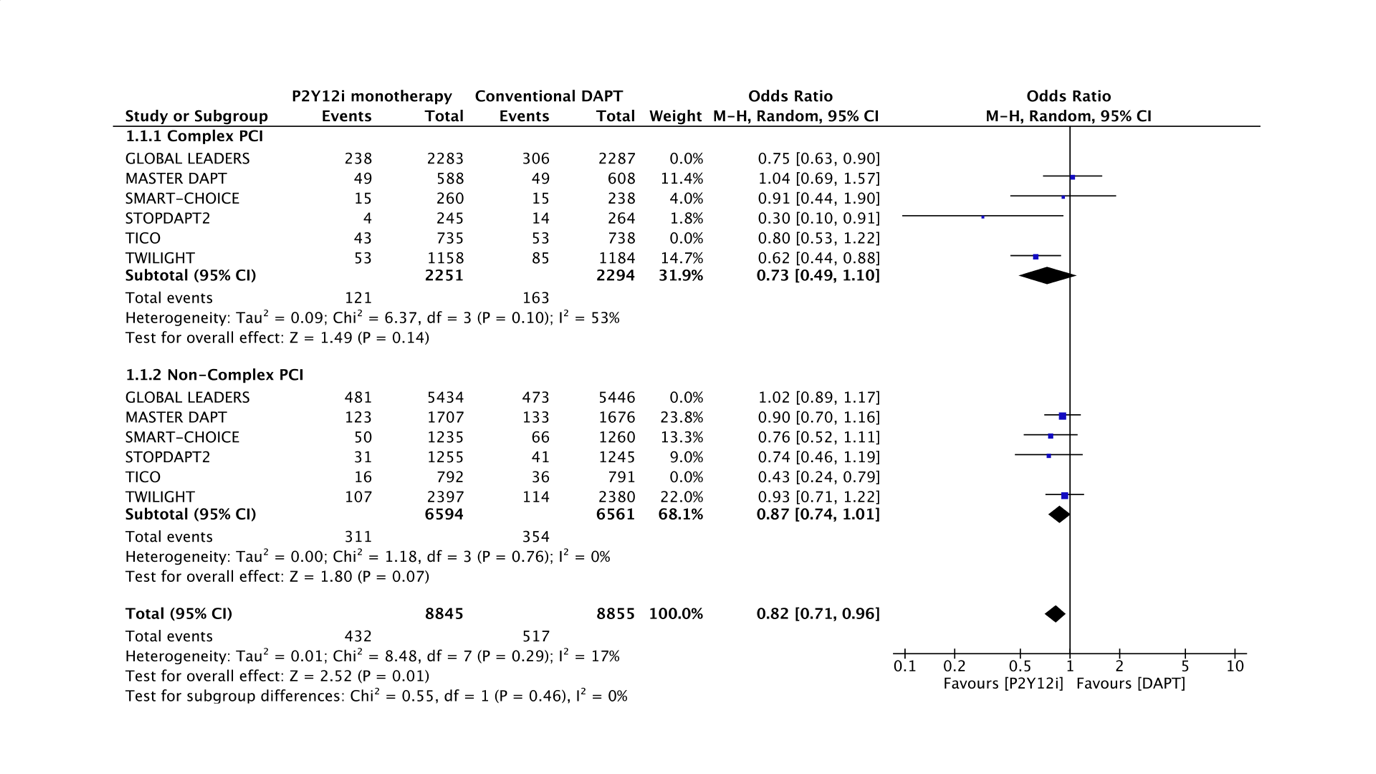
**

**Supplemental Figure 10. Risk estimations for MACCE without GLOBAL LEADERS and TICO**

**
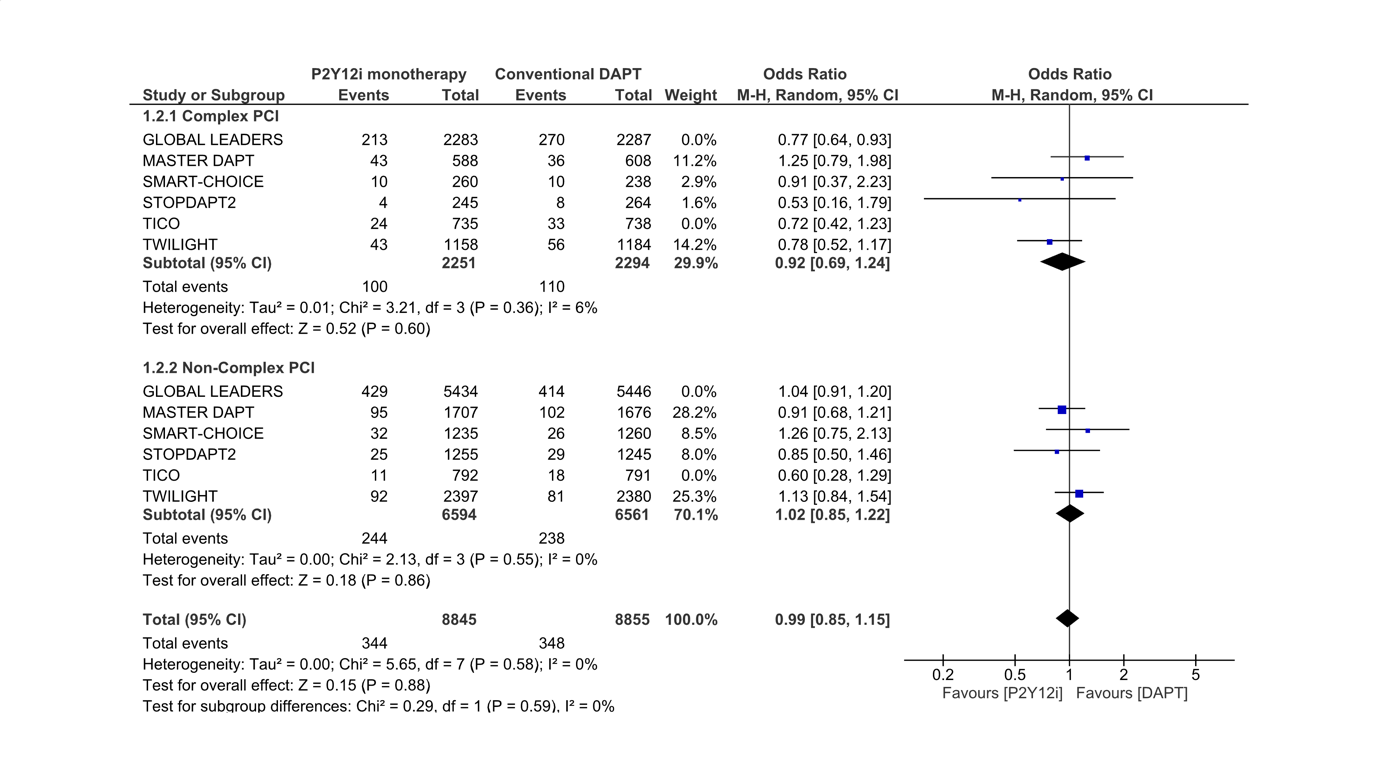
**

**Supplemental Table 1. Study characteristics**

| **Trial name** | **Blind** | **Study Population** | **Intervention** | **Control** | **Sample Size** | **Original primary endpoint** | **Follow-Up time** |
| --- | --- | --- | --- | --- | --- | --- | --- |
| **GLOBAL LEADERS^1^** | Open label | ACS (47%) or stable CAD after DES | Ticagrelor monotherapy after 1 month | Clopidogrel (stable CAD) or ticagrelor (ACS) + ASA 75 to 100mg daily | 15,968 | All-cause death or new Q-wave MI at 2 years | 24 months |
| **SMART CHOICE^2^** | Open label | ACS (58%) or stable CAD after DES | AnyP2Y12inhibitor monotherapy after 3 months | AnyP2Y12 inhibitor + ASA 100mg daily | 2,993 | Efficacy endpoint: MACCE (all-cause death, myocardial infarction, stroke); Safety endpoint: bleeding BARC 2-5 | 12 months |
| **STOPDAPT-2^3^** | Open label | ACS (38%) or stable CAD after DES | Clopidogrel monotherapy after 1 month | Clopidogrel + ASA 81 to 200mg daily after 1 month | 3,045  (3,009 in ITT) | Death from cardiovascular cause, MI, definite stent thrombosis, ischemic or haemorrhagic stroke, bleeding defined as TIMI major or minor criteria | 12 months |
| **TWILIGHT^4^** | Double blind | NSTE-ACS or stable CAD after DES | Ticagrelor monotherapy after 3 months | Ticagrelor + ASA81 to 100mg daily | 7,119 | BARC 2,3,5 bleeding (15 months after procedure) | 15 months |
| **TICO^5^** | Open label | ACS (STEMI or NSTE-ACS) after DES | Ticagrelor monotherapy after 3 months | Ticagrelor + ASA 100mg daily | 3,056 | MACCE (all-cause death, myocardial infarction, stent thrombosis, stroke, target vessel revascularization) | 12 months |
| **MASTER DAPT^6^** | Open label | ACS (49%) or stable CAD after DES | *ASA 75 to 162mg daily or any P2Y12 inhibitor after 1 month | †Any P2Y12 inhibitor after month 1 to 6-12 + ASA 75 to 162mg daily | 4,579 | NACE (death from any cause, myocardial infarction, stroke, major bleeding (BARC 3,5)); MACCE (death from any cause, myocardial infarction, stroke), major or clinically relevant non-major bleeding (BARC 2,3,5)) | 12 months |

*Patients on OAC used aspirin or clopidogrel with OAC after month1 to 6, OAC alone after 6 months.

†Patients on OAC used aspirin and clopidogrel with OAC after month1 to 3-12, either aspirin or clopidogrel with OAC was continued until 12 months. Abbreviations: ASA, acetylsalicylic acid; ACS, acute coronary syndrome; BARC, bleeding academic research consortium; CAD, coronary artery disease; DES, drug-eluting stent; MACCE, major adverse cardiac and cerebrovascular events; MI, myocardial infarction; NACE, net adverse clinical event; NSTE-ACS, non-ST-elevation acute coronary syndrome; STEMI, ST-elevation myocardial infarction.

**Supplemental Table 2. Risk of bias assessment**

|  | Random sequence generation (selection bias) | Allocation concealment (selection bias) | Blinding of participants and researchers (performance bias) | Blinding of outcome assessment (detection bias) | Incomplete outcome data (attrition bias) | Selective reporting (reporting bias) | Other bias |
| --- | --- | --- | --- | --- | --- | --- | --- |
| GLOBAL LEADERS | Low | Low | Unclear | Unclear | Low | Unclear | High* |
| SMART CHOICE | Low | Low | Unclear | Unclear | Low | Low | Low |
| STOPDAPT-2 | Low | Low | Unclear | Unclear | Low | Low | Low |
| TWILIGHT | Low | Low | Low | Low | Low | Low | Low |
| TICO | Low | Low | Unclear | Unclear | Low | Low | Low |
| MASTER DAPT | Low | Low | Unclear | Unclear | Low | Low | High† |

*Stable coronary artery disease patients in GLOBAL LEADERS varied between treatment arms both by choice of P2Y12 inhibitor and presence of aspirin. Although this decision was by experimental design, it introduces risk of bias in terms of interpretability of study results since two treatment factors differed between arms for this trial. †Patients with oral anticoagulants in MASTER DAPT varied between both arms by choice of oral anticoagulants and length of DAPT.

**References**

1 Serruys, P. W. *et al.* Impact of long-term ticagrelor monotherapy following 1-month dual antiplatelet therapy in patients who underwent complex percutaneous coronary intervention: insights from the Global Leaders trial. *Eur Heart J* **40**, 2595-2604, doi:10.1093/eurheartj/ehz453 (2019).

2 Roh, J. W. *et al.* P2Y12 inhibitor monotherapy in complex percutaneous coronary intervention: A post-hoc analysis of SMART-CHOICE randomized clinical trial. *Cardiol J* **28**, 855-863, doi:10.5603/CJ.a2021.0101 (2021).

3 Yamamoto, K. *et al.* Very Short Dual Antiplatelet Therapy After Drug-Eluting Stent Implantation in Patients Who Underwent Complex Percutaneous Coronary Intervention: Insight From the STOPDAPT-2 Trial. *Circ Cardiovasc Interv* **14**, e010384, doi:10.1161/CIRCINTERVENTIONS.120.010384 (2021).

4 Dangas, G. *et al.* Ticagrelor With or Without Aspirin After Complex PCI. *J Am Coll Cardiol* **75**, 2414-2424, doi:10.1016/j.jacc.2020.03.011 (2020).

5 Lee, S. J. *et al.* Ticagrelor Monotherapy Versus Ticagrelor With Aspirin in Acute Coronary Syndrome Patients With a High Risk of Ischemic Events. *Circ Cardiovasc Interv* **14**, e010812, doi:10.1161/CIRCINTERVENTIONS.121.010812 (2021).

6 Valgimigli, M. *et al.* Duration of Antiplatelet Therapy After Complex Percutaneous Coronary Intervention In Patients at High Bleeding Risk: a MASTER DAPT trial sub-analysis. *Eur Heart J*, doi:10.1093/eurheartj/ehac284 (2022).
